# Péricardite récidivante révélatrice d'un angiosarcome cardiaque

**DOI:** 10.11604/pamj.2015.21.97.7153

**Published:** 2015-06-08

**Authors:** Rim Klii, Wafa Chebbi

**Affiliations:** 1 Service de Médecine Interne et d'Endocrinologie, CHU Fattouma Bourguiba Monastir, 5000 Monastir, Tunisie; 2 Service de Médecine Interne, CHU Taher Sfar Mahdia, 5100 Mahdia, Tunisie

**Keywords:** Péricardite récidivante, tumeurs cardiaques, angiosarcome, Recurrent pericarditis, cardia tumor, angiosarcoma

## Image en medicine

Les tumeurs cardiaques primitives sont rares; leur incidence dans les séries d'autopsies varie de 0,001 à 0,03%, les trois quart sont bénignes (50% myxomes). La majorité des tumeurs malignes est représentée par des sarcomes ou des lymphomes cardiaques primitifs. L'angiosarcome est la tumeur maligne primitive du c'ur la plus fréquente (40%), localisée dans la plupart de cas dans l'oreillette droite, et plus fréquente chez l'homme. Les manifestations cliniques sont très variables, en rapport avec la localisation, la taille et l'extension de la tumeur et surviennent en général tardivement. Nous rapportons l'observation d'un angiosarcome cardiaque révélé par une péricardite récidivante. Il s'agissait d'un patient âgé de 53 ans, hospitalisé pour exploration d'une péricardite récidivante (3^ème^ épisode). A l'examen clinique, il présentait une masse thoracique gauche ferme et douloureuse à la palpation. L’échocardiographie montrait un ventricule gauche de taille normale et de fonction systolique conservée avec un décollement péricardique minime de 7-8 mm en postérieur et retro-auriculaire gauche. Le scanner thoracique objectivait un volumineux processus tissulaire de la cavité péricardique prolabé à l'oreillette droite, associé à une 2^ème^ masse pleuro pariétale médio-thoracique gauche avec en regard une lyse de la jonction chondro-costale. La biopsie écho-guidée concluait à un angiosarcome. L’évolution était marquée par une aggravation rapide sur le plan clinique et échographique. Le patient était transféré au service de radiothérapie pour un traitement palliatif.

**Figure 1:**
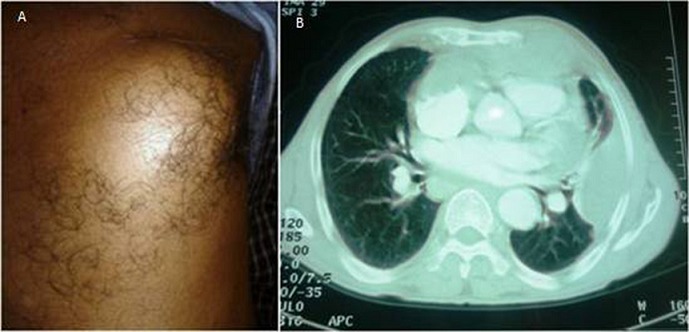
A) masse thoracique gauche; B) TDM thoracique coupe axiale: volumineux processus tissulaire de la cavité péricardique prolabé à l'oreillette droite, associé à une 2^ème^ masse pleuro pariétale médio-thoracique gauche avec en regard une lyse de la jonction chondro-costale

